# Oral Vitamin D Supplementation and Clinical Outcomes of Intravitreal Bevacizumab Injection for Macular Edema Secondary to Retinal Vein Occlusions

**DOI:** 10.18502/jovr.v17i3.11575

**Published:** 2022-08-15

**Authors:** Saeed Karimi, Farhad Parvizi, Amir Arabi, Toktam Shahraki, Sare Safi

**Affiliations:** ^1^Ophthalmic Research Center, Research Institute for Ophthalmology and Vision Science, Shahid Beheshti University of Medical Sciences, Tehran, Iran; ^2^Department of Ophthalmology, Torfeh Medical Center, Shahid Beheshti University of Medical Sciences, Tehran, Iran; ^3^Ophthalmic Epidemiology Research Center, Research Institute for Ophthalmology and Vision Science, Shahid Beheshti University of Medical Sciences, Tehran, Iran

**Keywords:** 25-Hydroxyvitamin D, Bevacizumab, Insufficiency, Intravitreal, Macular Edema, Retinal Vein Occlusion

## Abstract

**Purpose:**

To evaluate the therapeutic response of retinal vein occlusion (RVO) to intravitreal bevacizumab (IVB) with and without concomitant vitamin D supplementation.

**Methods:**

Seventy eyes of 68 patients with macular edema associated with branch retinal vein occlusion (BRVO) and central retinal vein occlusion (CRVO) received three monthly IVB injections. Patients with serum 25-hydroxyvitamin D (25(OH) D) higher than 30 ng/ml were considered as the sufficient group. Cases with serum 25(OH) D levels below 30 ng/ml were randomized into the treatment and control groups. The control group received 50,000 IU of oral vitamin D, weekly for two months. One month after the last IVB injection, best-corrected visual acuity (BCVA) and central macular thickness (CMT) were measured and compared with the preinjection values.

**Results:**

While 43 eyes (61.4%) of 42 patients had BRVO, 27 eyes (38.6%) of 26 patients had CRVO. In BRVO patients, changes of CMT and BCVA were not significantly different between the sufficient, control, and treatment groups (*P* = 0.58 and 0.64, respectively). In the CRVO group, CMT reduction in the control group was significantly less than the sufficient and treatment groups (*P* = 0.048). In addition, improvement of BCVA in the control group was significantly less (*P* = 0.036) than the sufficient and treatment groups.

**Conclusion:**

Oral vitamin D supplement therapy may improve anatomical and functional outcomes in patients with CRVO and vitamin D deficiency.

##  INTRODUCTION

Retinal vein occlusion (RVO) is a major cause of vision loss worldwide. Based on the location of vascular occlusion, RVO can be manifested as branch retinal vein occlusion (BRVO), central retinal vein occlusion (CRVO), or hemi-CRVO.^[1]^ In CRVO, the blockage occurs in the main retinal vein, whereas a BRVO is begun by an occlusion in smaller veins, mainly at arteriovenous crossovers through the retinal circulation. Macular edema and macular ischemia are the main causes of visual impairment in RVO, which are more frequent and less responsive to treatment in CRVO.^[1]^

Vitamin D is a nutritional supplement which plays an important role in various pathways in the body through the presence of its receptor in several tissues, including the bones, vascular myocytes, cardiac cells, hepatocytes, and immune cells. Recently, the role of this vitamin in vascular system health has been established through various studies.^[2]^ Both animal models and human studies have shown a positive correlation between vitamin D insufficiency and hypertension, vascular events, and mortality.^[3,4]^

A few studies have evaluated the relationship between different kinds of RVO and vitamin D insufficiency.^[1,5,6]^ The results of these studies suggest the role of vitamin D in preventing ocular vascular diseases. In the present study, in addition to the prevalence of vitamin D insufficiency in RVO cases, we assessed the effects of supplementing oral vitamin D on the efficacy of intravitreal bevacizumab (IVB) injections in RVO.

##  METHODS

The current prospective, interventional, single-center, randomized comparative study was carried out at a tertiary care center in Tehran between March 2018 and February 2019. The study was approved by the Research Ethics Committees of School of Medicine, Shahid Beheshti University of Medical Sciences, Tehran, Iran. The study followed the tenets of the Declaration of Helsinki. Written consent was obtained from all of the patients.

Patients with center-involving macular edema secondary to perfused BRVO or non-ischemic CRVO with an onset of less than three months prior were enrolled in the study. The diagnoses of RVO were made clinically by a single ophthalmologist (SK). A diagnosis of center-involving macular edema was made if the retinal thickness within central 1-mm of macula was > 300 μm as shown on the optical coherence tomography (OCT) image (Spectralis OCT, Heidelberg Engineering). The eyes received treatment if the BCVA was between 20/40 and 20/320 using the Snellen. The exclusion criteria were the following: age less than 18 years; patients on vitamin D supplementation or therapeutic diets; history of intravitreal anti-VEGF injections for the studied eye in the last three months of enrolment; history of intraocular surgery on the studied eye other than uncomplicated surgery for senile cataract; eyes with proliferative diabetic retinopathy or diabetic macular edema; and patients with renal, hepatic, and skin disease or chronic alcoholism.

All patients received IVB (AvastinⓇ) three times monthly. All injections were performed at the Torfeh Eye Hospital. During the administration of the injections, ophthalmologists were masked to the details of the study groups.

Demographic data and clinical parameters including BCVA and central macular edema (CMT) were measured for each subject. Visual acuity measurements were obtained through the Snellen chart examination by a trained optometrist who was masked to the study groups. Before the first injection, 25(OH) D was measured in venous blood samples. Patients whose measurements of 25-hydroxyvitamin D revealed to be > 30 ng/ml were considered as the vitamin D-sufficient group, while those with < 30 ng/ml were randomly assigned to the control and the treatment groups through simple consecutive randomization. There were three groups: group 1 (serum vitamin D ≥ 30 ng/ml), group 2 (vitamin D-insufficient group treated with vitamin D), and group 3 (control group). The treatment group received 50,000 IU of vitamin D3 weekly for eight weeks. For patients with bilateral RVO and macular edema (two patients), both eyes were enrolled in the same study group.

Follow-up examination and OCT imaging were performed one month after the completion of the IVB injection protocol. The mean changes in visual acuity and macular thickness were considered as primary and secondary outcomes, respectively. Oral vitamin D supplementation was prescribed for the control group at the end of the study protocol.

### Statistical Analysis

**Figure 1:**
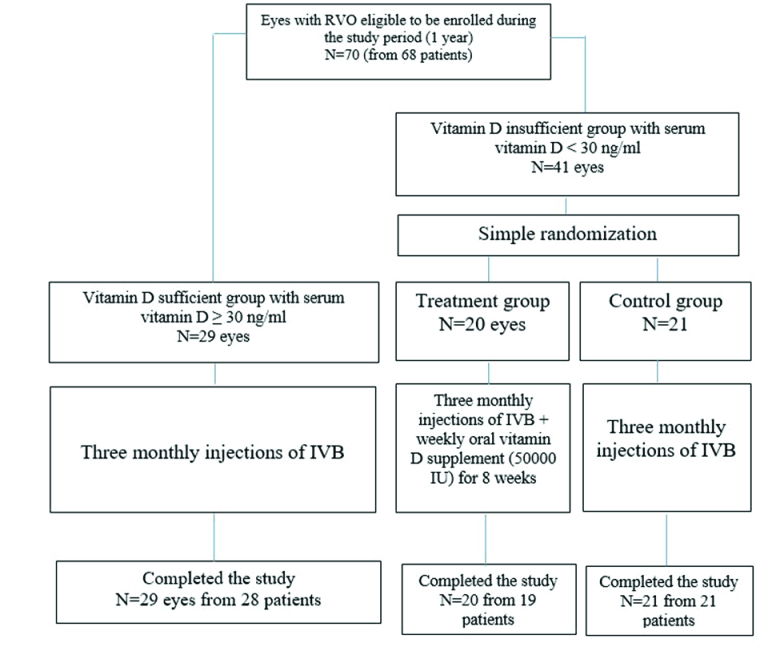
Flowchart of patient's enrollment. IVB, intravitreal bevacizumab.

**Table 1:** Characteristics of studied patients.


**Parameter**		**CRVO**			***P*** **-value**	**BRVO**			***P*** **-value**	**P-RVO**
		**Sufficient**	**Control**	**Treatment**	**Sufficient**	**Control**	**Treatment**	
Age	60.5 ± 7.81	56.57 ± 5.06	58.38 ± 9.69	0.571***	63.41 ± 6.94	65.5 ± 5.49	60.92 ± 9.81	0.305***	0.017**
25(OH)D level	40.31 ± 12.74	18.3 ± 7.83	19.49 ± 6.32	< 0.001***	40.68 ± 15.76	23.65 ± 4.72	18.07 ± 8.49	< 0.001***	0.913**
Gender	Male	8 (66.7%)	6 (85.7%)	6 (85%)	0.461*	7 (44%)	5 (35.7%)	6 (50.0%)	0.761*	
	Female	4 (33.3%)	1 (14.3%)	1 (15%)	9 (56%)	9 (64.3%)	6 (50.0%)	
Eye	OD	7 (58.3%)	2 (28.6%)	3 (37.5%)	0.405*	7 (41.2%)	8 (57.1%)	4 (33.3%)	0.452*	
	OS	5 (41.7%)	5 (71.4%)	5 (62.5%)	10 (58.8%)	6 (42.9%)	8 (66.7%)	
* According to Chi-square test and Fisher exact test; **According to *t*-test; ***According to ANOVA (Bonferroni for pairwise comparison); CRVO, central retinal vein occlusion; BRVO, branch retinal vein occlusion.										

**Table 2:** The correlation between vitamin D levels, BCVA and CMT.


		**Baseline BCVA in BRVO eyes**	**Baseline BCVA in CRVO eyes**	**Baseline CMT in BRVO eyes**	**Baseline CMT in CRVO eyes**
Vitamin D serum level	Pearson's correlation	–0.042	0.083	0.101	–0.022
	*P*-value	0.715	0.685	0.541	0.741
BCVA, best-corrected visual acuity; CMT, central retinal thickness; CRVO, central retinal vein occlusion; BRVO, branch retinal vein occlusion.					

**Table 3:** Changes of CMT following IVB in BRVO subgroups.


**CMT**	**BRVO Group**			***P*** -value*
	**Sufficient**	**Treatment**	**Control**	
Baseline CMT	469.82 ± 139.2	520 ± 182.03	491.33 ± 165.93	0.578
CMT at 3-month visit	355.88 ± 113.06	339.14 ± 101.87	366.83 ± 90.02		
Change of CMT	–126.44 ± 128.17	–180.86 ± 178.25	–124.5 ± 175.9		
*P*-value**	< 0.001	< 0.001	0.007		
*According to ANOVA analysis; **According to paired *t*-test; CMT, central retinal thickness; BRVO, branch retinal vein occlusion.				

**Table 4:** The Changes of BCVA following three IVB injections in BRVO subgroups.


**BCVA (Log MAR)**	**BRVO Group**			***P*** -value**
	**Sufficient**	**Treatment**	**Control**	
Baseline BCVA	0.6 ± 0.33	0.55 ± 0.45	0.57 ± 0.48	0.64
BCVA at 3-month visit	0.47 ± 0.34	0.29 ± 0.19	0.39 ± 0.28		
Change of BCVA	–0.14 ± 0.16	–0.26 ± 0.26	–0.17 ± 0.38		
*P*-value*	0.03	0.0179	0.179		
*According to paired *t*-test; **According to ANOVA; BCVA, best-corrected visual acuity; BRVO, branch retinal vein occlusion.				

**Table 5:** Changes of CMT following three IVB injections in CRVO subgroups groups.


**CMT**	**CRVO Group**			***P*** -value*
	**Sufficient**	**Treatment**	**Control**	
Baseline CMT	592.42 ± 272.63	575.14 ± 198.41	596.5 ± 248.69	0.047
CMT at 3-month visit	413.58 ± 190.3	403.29 ± 190.92	455.75 ± 97.26		
Change of CMT	–178.83 ± 216.64	–171.86 ± 112.69	–141.75 ± 290.98		
*P*-value**	0.0012	0.0039	0.016		
*According to ANOVA analysis; **According to paired *t*-test; CMT, central retinal thickness; CRVO, central retinal vein occlusion.				

**Table 6:** Improvement of BCVA following three IVB injections in CRVO subgroups.


**BCVA (Log MAR)**	**CRVO Group**			***P*** -value*
	**Sufficient**	**Treatment**	**Control**	
Baseline BCVA	0.94 ± 0.6	1.11 ± 0.69	1.1 ± 0.43	0.035
BCVA at 3-month visit	0.64 ± 0.4	0.69 ± 0.42	1.01 ± 0.31		
Change of BCVA	–0.3 ± 0.26	–0.42 ± 0.57	–0.09 ± 0.72		
*P*-value**	0.042	0.0236	0.844		
*According to paired *t*-test; **According to ANOVA; BCVA, best corrected visual acuity; CRVO, central retinal vein occlusion.				

To present data, mean and standard deviation were used. Chi-square and Fisher exact tests were used to compare between qualitative data. T-test and ANOVA (Bonferroni for pairwise comparison) were used for comparing quantitative parameters among the three study groups. To measure the role of treatment on BCVA and CMT, we used paired *t*-test analysis. The differences were considered as significant if p-value was < 0.05. All statistical analyses were performed by SPSS (IBM Corp. Released 2017. IBM SPSS Statistics for Windows, Version 24.0. Armonk, NY: IBM Corp.).

##  RESULTS

Seventy eyes of 68 patients (55.7% male and 44.3% female) were enrolled in this study. The mean age of the patients was 62 ± 8 years. While 43 eyes (61.4%) of 42 patients were diagnosed with BRVO, 27 eyes (38.6%) of 26 patients had CRVO [Figure 1].

The difference between the mean age of patients in the sufficient, control, and treatment groups was not statistically significant (mean difference = 2.11, *P* = 0.571, and 95% CI [0.65–5.28] for CRVO, mean difference = 1.98, *P* = 0.305, and 95% CI [0.31–7.1] for BRVO). The difference between the gender of patients and laterality of the affected eyes was also not statistically significant between these groups (*P* > 0.05). The mean age of BRVO and CRVO patients was 63.40 ± 7.49 and 58.85 ± 7.73 years, respectively, and the difference was statistically significant (mean difference = 5.81, *P* = 0.017, and 95% CI [3.69–9.37]) [Table 1].

While 28 patients had a sufficient level of 25(OH) D before the initiation of treatment, 40 patients had vitamin D deficiency. The prevalence of vitamin D deficiency in our study was 58.8%. Compared to the prevalence of vitamin D deficiency in Iranian population (55%),^[7]^ the prevalence of vitamin D deficiency in our RVO patients was not significantly different (*P* > 0.05).

Twenty-six eyes (60.4%) from the BRVO group and fifteen (27.2%) eyes from the CRVO group had insufficient levels of vitamin D. For both the groups, the prevalence of vitamin D deficiency was not significantly different from the reported prevalence among the Iranian population. Additionally, the levels of serum vitamin D were not significantly different between BRVO and CRVO patients (mean difference = 0.65, *P* = 0.25 and 95% CI [0.15–0.82]).

There was no significant correlation between serum vitamin D levels and BCVA or CMT at baseline [Table 2].

The changes of CMT following three monthly IVB injections were statistically significant in all BRVO subgroups. There were no significant differences in CMT changes among the “sufficient”, “control”, and “treatment” groups [Table 3].

Table 4 shows the changes of BCVA following three IVB injections in BRVO patients. The improvement of BCVA in the “sufficient” and “treatment” groups were statistically significant, but for the “control” group, this improvement was not statistically significant (*P* = 0.179). Changes of BCVA were not significantly different among the “sufficient”, “control”, and “treatment” groups (mean difference = 0.06, *P* = 0.64, and 95% CI [0.01–0.13]).

Three monthly IVB injections significantly decreased CMT in all CRVO subgroups (*P* < 0.05). However, the decrement of CMT in the control group was less than the “sufficient” and “treatment” groups, and the difference among CMT changes in the CRVO subgroups was statistically significant (*P* = 0.047) [Table 5].

In CRVO patients, improvement of BCVA following three IVB injections was statistically significant in the “sufficient” and “treatment” subgroups (*P* < 0.05); however, in the “control” group, change of BCVA was not statistically significant (*P* = 0.84). Improvement of BCVA in the “control” group was significantly less than the “sufficient” and “treatment” groups (*P* = 0.035) [Table 6].

##  DISCUSSION

In the present study, although the prevalence of vitamin D deficiency in patients with BRVO and CRVO was higher than the overall prevalence of vitamin D deficiency in the Iranian population,^[7]^ the difference was not statistically significant. We did not find significant correlations between serum vitamin D levels and their effect on the baseline BCVA and CMT measurements in BRVO and CRVO patients. In the BRVO subgroups, vitamin D supplement therapy did not have significant influence on anatomical and functional outcomes of the IVB injections. In CRVO subgroups, however, vitamin D supplement therapy had significant beneficial effects on both anatomical and functional outcomes of the IVB injections. The changes of BCVA and CMT following three IVB injections were lower in patients with CRVO and vitamin D insufficiency who were not treated with oral vitamin D supplement therapy.

Vitamin D, whether as a nutritional supplement or as a hormone, has numerous roles to play.^[8]^ It participates in the synthesis of many factors which are involved in various metabolic mechanisms other than calcium homeostasis. Vitamin D insufficiency can be caused by limited UV exposure, limitations of dietary intake, and by numerous chronic skin and internal diseases. Through its dependence on sunlight exposure and dietary habits, serum levels of vitamin D may be affected by some cultural and geographic issues.^[9]^ Recently, a positive correlation has been postulated between vitamin D insufficiency and the incidence of different vascular diseases. This correlation has been established for cardiac and cerebrovascular diseases, as well as hypertension.^[10]^ Both epidemiologic and observational studies have reported higher mortality due to vascular events in vitamin D-deficient patients, which may happen during winter and in regions with less UV-B exposure.^[11]^ Low serum vitamin D levels may be a risk factor for vascular diseases.^[12]^ It is notable that systemic vascular diseases and retinal vascular occlusions have some risk factors in common. A non-classical function of vitamin D, an improvement in the vascular endothelial function, has been reported following vitamin D supplement therapy.^[13]^ A meta-analysis reported that patients with type 2 diabetes mellitus and vitamin D deficiency have a higher risk of diabetic retinopathy.^[14]^ Additionally, it is believed that vitamin D plays a critical role in the modulation of the immune system. It has been suggested that 25(OH) D dampens the activation of cytokines and reduces the proliferation of inflammatory cells.^[15]^

Vitamin D has received attention for its role in reducing vascular events, regulating the renin-angiotensin system and endothelial hemostasis, controlling coagulation, and promoting anti-inflammatory properties.^[16]^ The role of 25(OH) D deficiency in systemic vascular risks, endothelial homeostasis, and inflammatory conditions could possibly relate to the pathogenesis of RVO.

After diabetic retinopathy, RVO is considered as the most frequent retinal vascular disease.^[17]^ Macular edema secondary to venous occlusion is the most common cause of visual loss in RVO patients.^[18]^ It can happen via inflammatory mechanisms, vascular endothelial growth factor production, or mechanical effects of increased intraluminal pressure. Atherosclerotic and vascular risk factors, including hypertension and hyperlipidemia, have been established to be involved in RVOs.^[18]^ Similarly, the role of inflammation in progression and complications of retinal diseases, such as RVO, has been recognized.^[19]^ The potential role of thrombophilia in RVO has also drawn attention during recent years.^[20]^

According to a recent study by Epstein et al, vitamin D is deficient in 50% of CRVO patients.^[5]^ Vitamin D deficiency had been previously reported in a case of CRVO.^[6]^ The results of a recent study on a subset of Indian patients pointed toward the role of vitamin D in ocular vascular mechanisms and retinal vascular occlusion.^[1]^ These studies have also suggested a seasonal variation for RVO.

In addition, recently, a probable positive role has been postulated for short-term vitamin D supplementation in reducing inflammatory and oxidative damage of the vascular system.^[21]^ To the best of our knowledge, this is the first study evaluating the role of vitamin D supplement therapy in anatomical and functional outcomes of IVB injections in RVO patients. For each RVO subgroup (BRVO and CRVO), the patients were categorized as vitamin D-sufficient versus -deficient. The anatomical effects of the IVB injections were statistically significant in all study groups. The improvement of BCVA following three IVB injections were not statistically significant in patients with BRVO or CRVO along with vitamin D insufficiency who were not treated with oral vitamin D supplement therapy. Additionally, in CRVO subgroups, a positive correlation was observed between 25(OH) D supplementation and better anatomical and functional outcome of IVB injections.

A small sample size is the main limitation of the present study. Another limitation existed where serum vitamin D levels were not re-measured throughout the study to confirm the efficacy of supplementation therapy. Finally, there may be some confounding factors such as different dietary intake of vitamin D among our patients along with seasonal variations during the study. Future randomized clinical trials with larger sample sizes are needed to reveal the exact role of vitamin D in RVO patients.

In summary, we observed that oral vitamin D supplement therapy significantly improved both the functional and anatomical outcomes of IVB injections in CRVO patients with vitamin D insufficiency. Patients receiving oral supplementation experienced more decrease in CMT and better improvement in BCVA following IVB therapy, compared to the control group. Oral vitamin D supplement therapy did not change the outcomes of IVB injections in the BRVO cases.

##  Ethical Approval

All procedures performed in the study were in accordance with the 1964 Helsinki Declaration. This study was approved by the Ethics Committee of the Ophthalmic Research Center at Shahid Beheshti University of Medical Sciences, Tehran, Iran.

##  Financial Support and Sponsorship

None.

##  Conflicts of Interest

None.
